# Diabetes policies and pharmacy-based diabetes interventions in Portugal: a comprehensive review

**DOI:** 10.1186/s40545-019-0166-1

**Published:** 2019-03-21

**Authors:** Suzete Costa, Maria Rute Horta, Rita Santos, Zilda Mendes, Isabel Jacinto, José Guerreiro, Maria Cary, Ana Miranda, Dennis K. Helling, Ana Paula Martins

**Affiliations:** 1 USFarmácia® Collaborative Care Project, Associação Nacional das Farmácias, R. Marechal Saldanha, 1, 1249-069 Lisboa, Portugal; 2 Department of Pharmacy Services, Associação Nacional das Farmácias, R. Marechal Saldanha, 1, 1249-069 Lisboa, Portugal; 3 Post-Graduate School of Health & Management (EPGSG), Associação Nacional das Farmácias, R. Marechal Saldanha, 1, 1249-069 Lisboa, Portugal; 4 Centre for Health Evaluation & Research (CEFAR), Associação Nacional das Farmácias, R. Marechal Saldanha, 1, 1249-069 Lisboa, Portugal; 5 Registo Oncológico Nacional, IPO Lisboa, R. Prof. Lima Basto, 1099-023 Lisboa, Portugal; 6 University of Colorado Skaggs School of Pharmacy and Pharmaceutical Sciences, 8189 East 5th Avenue, Denver, CO 80230 USA; 7 Research Institute for Medicines (iMed.ULisboa), Faculty of Pharmacy, Universidade de Lisboa, Lisboa, Portugal

**Keywords:** Review, Community pharmacy, Diabetes, Policies, Interventions, Pharmacy services, Public health, Portugal

## Abstract

**Background:**

Pharmacy-based interventions are complex public health endeavors which include, but are not restricted to, the conventional medication supply role. In diabetes, such interventions may improve patients’ outcomes. The aim of this study was to review relevant policies and research developed in Portugal directed at pharmacy-based diabetes interventions, and to inform future policies, practice and research in collaborative practice with primary care.

**Research method:**

An exploratory review of diabetes legislation and policy papers, as well as a comprehensive review in Embase, MEDLINE (via Ovid and PubMed), Google Scholar, and grey literature until November 2017 was performed.

**Results:**

Sixteen policy papers and 10 studies were included in the analysis. Positive evidence from pharmacy interventions was retrieved concerning screening individuals at risk, screening uncontrolled patients, managing diabetes, and supporting self-monitoring.

**Conclusions:**

Some consistency in favorable findings, but also room for improvements in health policies, intervention design and research methods, were observed.

**Electronic supplementary material:**

The online version of this article (10.1186/s40545-019-0166-1) contains supplementary material, which is available to authorized users.

## Background

A research study conducted in 2013 in the U.S. health system examined avoidable costs in six areas of “opportunity” [1]. Amongst six diseases, avoidable costs related with diabetes medication non-adherence represented 23.4% of the total non-adherence costs and second in rank. An opportunity of $39 billion US dollars of avoidable costs from delayed evidence-based treatment was estimated. Among four diseases analyzed, diabetes represented 90% of avoidable spending. The study further pointed out that improvements are possible only through collaboration between healthcare stakeholders: providers, pharmacists, patients, payers, pharmaceutical manufacturers and policymakers [1].

Patient care interventions provided by pharmacies can be defined as complex public health interventions which are beyond, but do not necessarily exclude, the medication supply role, usually provided by pharmacists to patients in the community pharmacy setting [2]. In diabetes, such interventions may include health promotion and diabetes prevention, screening of at-risk individuals, diabetes management (including medication management), patient education and support on self-monitoring, and medical referral, when appropriate.

Complex health interventions require several interacting components, including behavioral changes from providers and individuals, a coordinated action at multiple levels, and some degree of flexibility of interventions.

Strategies used to explain how pharmacy-based interventions might work seem to be consistent with public health theories, namely the Diffusion of Innovation Theory [3] to accelerate the adoption of important interventions by providers, the Theory of Planned Behavior [4] to explain patient decision of submitting to screening, the Social Cognitive Theory and the Transtheoretical Model [5] for behavioral strategies used in lifestyle modification interventions, and the Information-Motivational-Behavioral Skills Model [6] to guide strategies used in interventions directed at diabetes medication adherence.

Behavioral changes in pharmacists and patients can subsequently lead to improved health outcomes [7].

Evidence exists concerning improvements in health outcomes due to diabetes interventions provided by community pharmacists across different jurisdictions.

Mossialos et al. conducted an umbrella study to identify systematic reviews of effectiveness of community pharmacist interventions [8]. Ellitt et al. reviewed 21 studies evaluating the effectiveness of pharmacist interventions, with positive findings for patients with diabetes [9]. Collins et al. reviewed 14 randomized controlled trials (RCTs) and showed a positive and consistent association between pharmacist intervention and glycemic control improvement [10]. Machado et al. reviewed 36 studies and concluded that education and medication management interventions resulted in positive outcomes on hemoglobin A1c (HbA1c) and blood pressure [11]. Wubben et al. reviewed 21 studies that showed improvements in HbA1c [12]. Blenkinsopp et al. reviewed seven experimental studies, also with positive findings concerning HbA1c [13].

Rotta and colleagues sought to investigate the impact of clinical pharmacy services on medication use and patient outcomes [14]. Their overview included four systematic reviews assessing impact on diabetes management [11, 12, 15, 16]. All studies included patient education and counseling, therapy and lifestyle modifications, and showed positive results. HbA1c reduction ranged from 0.9 to 2.1%. Drug therapy adjustments after medication review and medication follow-up were also reported in two of the included reviews [11, 12].

Finally, Wang et al. conducted a systematic review of 10 community pharmacy studies which aimed to perform an economic evaluation of services provided and managed by pharmacists for people with diabetes [17]. The study addressed disease management and medication review and had a median follow-up of 12 months. Six of the included studies were full economic evaluations and four of them presented favorable results [18–21].

A study by Nathan et al. sought to determine the mathematical relationship between HbA1c and average glucose (AG) levels [22]. The linear regression equations between HbA1c and AG did not differ significantly across subgroups based on age, sex, diabetes type, race/ethnicity, or smoking status and the study concluded that HbA1c levels can be expressed as estimated AG for most patients with type 1 and type 2 diabetes [22]. This is relevant as most pharmacies perform blood glucose measurements.

In a list of 44 Organization for Economic Co-operation and Development (OECD) countries, Portugal ranks seventh in terms of diabetes prevalence in adults [23]. Prevalence of the condition in the country was estimated to be 9.9% in 2015 in adults aged 20–79 years old, which is higher than the OECD average and makes this issue a national health priority [23].

Torre et al. showed that the consumption of glucose lowering drugs (GLD) in Portugal increased 32% over a 10-year period between 2004 and 2013 [24], mainly due to the rapid uptake of fixed-dose combinations, mostly DPP-4 inhibitors, which represented almost a quarter of the total GLD consumption in 2013, in defined daily doses per 1000 inhabitants per day (DHD). This represents a higher consumption rate of fixed-dose combinations than the Netherlands and higher than eight European countries. In 2010, the market share of fixed-dose combinations in Portugal was six times higher than Denmark, nine times higher than the UK, and 13 times higher than Sweden in DHD. Conversely, Portugal was the country with the lowest intake of metformin alone (34.4% in 2010) [24].

Self-monitoring blood glucose (SMBG) strips are prescribed by physicians, reimbursed by the National Health System (NHS) and dispensed in pharmacies. Sales of SMBG strips increased markedly (over 70%) in Portugal between 2006 and 2015 [25]. In 2015, this represented a 44.7 million euro expenditure for the NHS (+ 3.8% versus 2014) [25].

Despite consumption patterns, only 37.5% of diabetic patients in Portugal have their disease controlled [26].

Portuguese pharmacies have a long history of public health interventions beyond only medication provision. Measurement of clinical parameters, administration of injectable medicines, treatment of minor wounds, and blood draw are authorized and have been performed in Portuguese pharmacies since 1968 [27].

In 1989, WHO Regional Office for Europe, in collaboration with the International Diabetes Federation (IDF) Europe, initiated the St. Vincent Declaration Action Program (SVD), supported by the Portuguese health authorities. Portuguese pharmacies embraced the initiative and started a structured intervention plan for diabetes patients, comprising regular monitoring of blood glucose and blood pressure. Pharmacists’ intervention also included diabetes early detection, patient information and education.

Several other structured pharmacy-based diabetes interventions took place in the following years, which are the object of this review.

## Review question

The present review aims to provide a historical perspective of the evolution of Portuguese health policies relevant for pharmacy-based diabetes interventions, to review diabetes interventions performed in Portuguese pharmacies over the last 25 years, and to inform future policies, practice and research about the effectiveness and value of pharmacy-based diabetes interventions under collaboration with primary care.

## Methods

### Literature search

A mixed method approach was used. An exploratory review of diabetes legislation and policy papers relevant to pharmacy-based diabetes interventions was performed. A comprehensive review was conducted on Embase (via Ovid), MEDLINE (via Ovid and PubMed), Google Scholar, authors’ personal files and grey literature through November 2017, to identify published and unpublished studies focusing pharmacy-based diabetes interventions performed in Portuguese pharmacies. Experts from academia were also contacted to identify additional studies.

Database search strategy consisted in an iterative process that used the terms “(((pharmacy OR pharmacist OR pharmaceutical) AND diabetes AND Portugal) NOT Hospital)”, with minor adjustments according to database specifications (Additional file 1).

Literature search was limited to English and Portuguese, and restricted to (i) title, original title, abstract, heading word, keyword, keyword heading word or text word in MEDLINE and Embase, (ii) title and abstract in PubMed, and (iii) title in Google Scholar.

### Study selection

Studies were included if they met the following inclusion criteria: being reviews or primary studies; focusing diabetes (exclusively or not); assessing the impact of patient care interventions; having been developed in the community pharmacy setting; having been developed in Portugal. Grey literature was limited to national, regional or group of pharmacies’ interventions. Grey literature of studies conducted in a single pharmacy/single owner was deliberately excluded. Since complex healthcare interventions are influenced by patient and pharmacist behaviour, pharmacies and the health system, the authors aimed to capture interventions that could reflect several types of pharmacies, owners, and staff pharmacists instead of just a single pharmacy/single owner initiative.

The study population consisted of diabetic patients or individuals at risk of diabetes.

Patient care interventions were defined as complex public health interventions, provided by pharmacists to patients in the community pharmacy setting, with the purpose of preventing disease, promoting health, and prolonging life (beyond, but not necessarily excluding, the medication supply role) [2].

No restrictions on comparators were considered.

Some of the outcomes analyzed following intervention included, but were not limited to, medication adherence changes, medication changes, blood glucose/HbA1c changes, patient reported outcome (PRO) changes, changes in patient quality of life (QoL) and satisfaction, proportion of at-risk patients, and economic outcomes.

Citations arising from the literature search were extracted and duplicates, removed. All potentially relevant titles were checked against inclusion criteria, and abstracts of retrieved titles were reviewed. Full-text articles of retrieved abstracts were also checked for eligibility. Reasons for study exclusion at screening were recorded.

The PRISMA Flowchart was used to report study selection [28].

A chronological narrative synthesis of policy papers and studies was performed.

## Results

The exploratory analysis identified 16 relevant legislation and policy papers focusing key events.

**Fig. 1:**
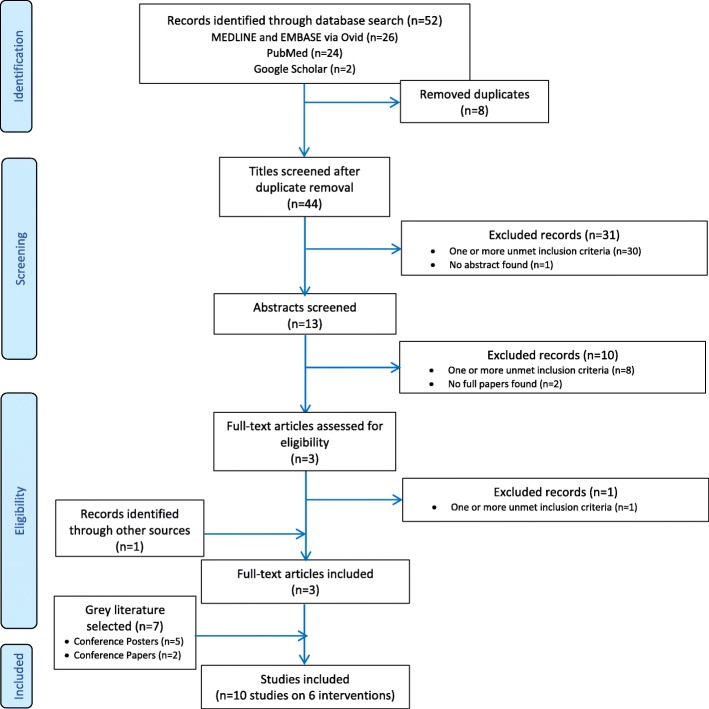
Study selection flowchart

Electronic literature search of published and unpublished studies initially retrieved 52 potential citations, eight of which were duplicates and removed, leaving 44 potential studies. Title screening excluded 31 studies, leaving 13 potentially relevant titles. Abstract assessment excluded 10 additional studies, leaving three potentially relevant abstracts. Full-text assessment retrieved three studies, to which grey literature added seven more studies. Overall, 10 studies were included in the analysis (Fig. 1).

Table 1 lists the legislation and policy papers included in the analysis and Table 2 describes the characteristics of the 10 studies included.

**Table 1:** List of legislation/policy papers included in the analysis

Reference	Policy paper
[29]	First European guidelines on pharmacy-based diabetes management, 1996
[30]	First Agreement on the National Program for Diabetes Control, 1998
[33]	Second Agreement on the National Program for Diabetes Control, 2003
[34]	Distribution and pricing of SMBG - Decree 509-B/2003, 30 June
[40]	Health interventions allowed in pharmacies – Decree 1429/2007, 2 November
[41]	Third Agreement on the National Program for Diabetes Control, 2008
[42]	Pricing of SMBG – Decree 253-A/2008, 4 April
[43]	Pricing and reimbursement of SMBG – Decree 364/2010, 23 June
[44]	Prescribing rules, prescription forms, dispensing and patient information rules – Decree 137-A/2012, 11 May
[45]	Price reduction of SMBG – Order 4294-A/2013, 22 March
[46]	First Framework Agreement for Community Pharmacy, 2014
[47]	Pricing and reimbursement of SMBG – Decree 222/2014, 4 November
[50]	Pricing and reimbursement of SMBG – Decree 35/2016, 1 March
[51]	Framework for pharmacy-based public health interventions and incentives for dispensing reimbursed medicines of reference price groups – Decree-Law 62/2016, 12 September
[53]	Second Framework Agreement for Community Pharmacy, 2017
[54]	Health interventions allowed in pharmacies – Decree 97/2018, 9 April

**Table 2:** Characteristics of the 10 studies included

First author (year of publication / presentation)	Pharmacy-based diabetes intervention level	Description of intervention	Study design	No. of pharmacies	No. of patients / sample size
Santos MR (2003) [31]	Diabetes management	Regular scheduled follow-up of diabetic patients using SOAP method, as per intervention protocol: Subjective: patient reported information; Objective: e.g. blood glucose measurements Assessment: Drug Related Problems (DRPs) Plan: e.g. referral to physician, patient education and information	Retrospective evaluation using historic pilot cohort Diabetes Management Program (2001)	31	143
Costa S (2006) [32]	Diabetes management	Regular scheduled follow-up of diabetic patients using SOAP method, as per intervention protocol: Subjective: patient reported information; Objective: e.g. blood glucose measurements Assessment: Drug Related Problems (DRPs) Plan: e.g. referral to physician, patient education and information	Retrospective evaluation using historic pilot cohort Diabetes Management Program (2001)	NR	NR
Martins AP (2008) [35]	Diabetes management	Regular scheduled follow-up of diabetic patients using SOAP method, as per intervention protocol: Subjective: patient reported information; Objective: e.g. blood glucose measurements Assessment: Drug Related Problems (DRPs) Plan: e.g. referral to physician, patient education and information	Retrospective study using historic 3-year cohort of the Diabetes Management Program (2003–2006)	NR	Around 1800 in program, 342 in study
Fernandez-Llimos F (2009) [36]	Diabetes management	Regular scheduled follow-up of diabetic patients using SOAP method, as per intervention protocol: Subjective: patient reported information; Objective: e.g. blood glucose measurements Assessment: Drug Related Problems (DRPs) Plan: e.g. referral to physician, patient education and information	Observational - process indicators of Diabetes Management Program (2004–2008)	356 in 2005 379 in 2008	1294 in 2005
Pilger D (2007) [37]	Screening of individuals at risk of diabetes	Screening patients with > 45 years and another risk factor for diabetes: blood glucose measurement and referral to physician as per intervention protocol	Descriptive cross-sectional study (2005)	8	229
Horta MR (2010) [38]	Screening of uncontrolled diabetic patients	Screening patients on diabetes medications: blood glucose measurement, reinforcing adherence and self-monitoring, and referral to physician as per intervention protocol	Descriptive cross-sectional evaluation of nationwide campaign (2007)	723	7719
ES Research (2009) [39]	Screening of uncontrolled diabetic patients	Screening patients on diabetes medications: blood glucose measurement, reinforcing adherence and self-monitoring, and referral to physician as per intervention protocol	Economic evaluation of nationwide campaign (2007)	723	7719
Jacinto I (2016) [48]	Screening of individuals at risk of diabetes	Promoting FINDRISK test to all adult non-diabetic individuals (except pregnant women) and healthy lifestyle habits, referral to physician if score > 15 points.	Descriptive cross-sectional evaluation of regional campaign (2015)	225	7007
Paulino E (2017) [49]	Screening of individuals at risk of diabetes and diabetes management	1) Promoting FINDRISK test to all individuals and referral of high-risk patients to pharmacy services, to physician or re-evaluation in 1 year, as appropriate 2) Follow-up of diabetic patients with assessment of health problems, reinforcing adherence, referral to other providers or to pharmacy consultations	Descriptive cross-sectional evaluation of regional initiative (2016/17)	1) 26 2) 31	1) 196 2) 106
Félix J 2017 [52]	Screening of individuals at risk, screening of uncontrolled diabetic patients, and diabetes management	Includes the interventions of studies [31, 32, 35, 38] *(Economic study partially based on data of these studies)*	Economic evaluation (decision-model)	NA (model-based)	NA (model-based)

*NR* not reported, *NA* not applicable

### Findings of the review

In 1993, Portugal was appointed to lead the EuroPharm Forum/WHO (Forum of European Pharmaceutical Associations and the World Health Organization European Region) Task Force on Pharmacy-Based Diabetes Management (PharmaDiaß Programme). This Task Force produced the first European guidelines on pharmacy-based diabetes management in 1996 [29].

In 1998, the First Agreement on the National Program for Diabetes Control was signed between the Portuguese Ministry of Health and several stakeholders for four years, aiming at improving self-monitoring of diabetic patients. According to this agreement, reimbursed SMBG products were to be dispensed in pharmacies for diabetic patients of the National Health Service (NHS), who no longer had to pay the total amount upfront and wait for the reimbursement. With the Agreement, pharmacies advanced this reimbursement on behalf of the NHS and diabetic patients had to pay only a small co-payment for the strips. Syringes, needles and lancets were free of charge. In addition, pharmacies were to provide specific counseling. Pharmacies agreed not to be remunerated for this service until December 1999 [30].

In 1999, the Portuguese National Association of Pharmacies (ANF) developed methods and tools for a pharmacy-based Diabetes Management Program in Portugal, based on the work developed by US clinical pharmacists from Kaiser Permanente Colorado Region and from CVS chain of pharmacies. Results of the 2001 pilot trial were used to adjust the model for an expansion phase in 2003 [31, 32].

The First Agreement ending in December 2002 was extended until June 2003 and was set to be revised on the terms of stakeholder collaboration aiming to define new intervention strategies.

In September 2003, a Second Agreement was forged with the Ministry of Health for a period of two years. This agreement foresaw, for the first time, payment for the provision of the Diabetes Management Program in pharmacies set at 12,00€ (euro) per patient per month, reimbursed in 75% by the NHS. It was the first capitated payment for Portuguese pharmacies to provide a full scope service [33].

At the same time, reimbursed SMBG products continued to be dispensed in pharmacies to diabetic patients of the NHS under a facilitated access scheme, with pharmacies having no profit margin [34].

An evaluation of this program was performed in 2006. A patient sub-cohort was analyzed, consisting of diabetic patients on antidiabetic medication, with blood glucose levels above target values, and with, at least, three blood glucose readings at baseline, 3, and 6 months. A total of 342 patients (out of approx. 1800 followed in pharmacies) were included (62.9% female), averaged 64.1 years old. Drug Related Problems (DRPs) were identified in 74% (*n* = 253) of patients, most of which (78.5%) were related to medication non-effectiveness and 18.1%, to non-adherence. Pharmacists referred 84% (*n* = 212) of patients with DRPs to the physician, which represented 63% (*n* = 403) of DRPs overall. Fifty three percent (*n* = 215) of reported DRPs resulted in therapy modification in 69% (*n* = 147) of patients. Blood glucose values at 3 and 6 months suggested a 13.5 mg/dL decrease in fasting blood glucose, a 34.0 mg/dL decrease in post-prandial blood glucose, a 7.99 mg/dL decrease in total cholesterol, a 3.39 mmHg decrease in systolic blood pressure, and a 0.7% decrease in HbA1c. Furthermore, 21% of initially uncontrolled diabetic patients reached target control values at 3 months and remained controlled at 6 months. For patients who did not achieve blood glucose targets at 6 months, a significant decrease in all parameters was still observed [35].

The outreach of this program in terms of proportion of participating pharmacies and patients at municipality level was assessed in 2009. The authors considered that, although positive effects on diabetes management were observed with the program, its implementation was too low and could not be considered successful. They recommended a new analysis on implementation barriers and facilitators in pharmacies [36].

A group of investigators assessed the applicability of a protocol designed to identify undiagnosed patients with type 2 diabetes and associated risk factors in eight pharmacies. Pharmacists assessed 229 patients presenting risk factors for the condition, 14% of which were identified and referred to the physician. A diagnosis was retrieved by 68.7% of pharmacists, resulting in 12 diagnosed patients [37].

In 2007, a nationwide diabetes campaign was implemented in pharmacies for 6 days to identify uncontrolled diabetic patients [38]. Overall, 723 pharmacies assessed 7719 diabetic patients (57.2% female). Average age was 66 years and 36.2% had a Body Mass Index (BMI) > 30 kg/m^2^. The majority (87.3%) of patients were on oral antidiabetic medications, 7.6% were on insulin, and 5.1% were on combination therapy (insulin and oral antidiabetic medication). Pharmacists performed a total of 11,102 blood glucose readings. Average postprandial blood glucose was 189.5 mg/dL and average fasting blood glucose was 144.8 mg/dL. For 47% of patients, postprandial blood glucose was > 180 mg/dL or fasting blood glucose was > 130 mg/dL. Compared to patients on insulin only, those on oral antidiabetics had a higher probability of being within the recommended blood glucose values (odds ratio [OR] = 1.297; 95% confidence interval [CI], 1.061–1.587). A referral to the prescriber was reported for 23.9% of patients, of which 72.7% had blood glucose above the recommended values [38].

An economic evaluation of this campaign estimated that pharmacists’ interventions resulted in 4-million-euro cost savings [39].

In 2007, the legislation responsible for defining the scope of health interventions in pharmacies included, for the first time, disease management programs. Although without specifying diabetes, these included the Diabetes Management Program, already in place since 2001 and remunerated since 2003 [40].

In 2008, the Third Agreement was forged with the Ministry of Health for another two years, amending upwards the remuneration for pharmacies’ provision of the Diabetes Management Program for 15,00€ (euro) per patient per month [41].

At the same time, prices of SMBG products were revised and continued to be dispensed in pharmacies to the NHS diabetic patients on a facilitated access scheme, with no profit margin for pharmacies [42].

Despite positive results, the Third Agreement ceased in 2010 as per Government’s decision, ending the first experience of remunerating pharmacies for a full scope patient care service. In addition, although SMBG products continued to be dispensed in pharmacies, product margins ceased to be regulated to be freely negotiated between each pharmacy and the industry. However, the new policy stated that a working group was to be defined to present a proposal for a new pricing and margin methodology [43].

In 2012, under an austere economic environment, stringent rules for prescribing and dispensing medicines were adopted, namely concerning patient’s right to choose one commercial brand within medications’ International Nonproprietary Names (INN). New rules were set to be applied also to reimbursed products, namely SMBG products [44]. In 2013, prices for these products were cut by 15% [45], although the legislation was later revoked.

In 2014, the first Framework Agreement for Community Pharmacy was signed with the Ministry of Health. This Agreement established, for the first time, directives for the development of public health programs in Portuguese pharmacies and incentives for generic dispensing. Priority areas included self-monitoring of diabetes and adherence monitoring. The Agreement foresaw economic evaluation of pilot trials and payment of interventions with proven effectiveness and cost-effectiveness. The Agreement ended in December 2015, after the Government’s term of office came to an end, with no pilot study on diabetes self-monitoring or adherence monitoring, despite advances in needle exchange and generic incentives [46].

Later that year, prices of SMBG products were reduced, keeping the same policy of unregulated margins for pharmacies regarding product dispensing [47].

Despite these policies, in 2015, 225 pharmacies participated in the November World Diabetes Month campaign in the Centre Region of Portugal, aimed at identifying individuals at risk of diabetes. A total of 7007 patients (mean age 60 years, standard deviation [SD] = 14.97 years) were assessed, 79.31% of which were ≥ 45 years and 66.05%, female. A high percentage (66.61%) of overweight patients (BMI ≥ 25 Kg/m^2^) was observed. An also high percentage (81.31%) of women had waist circumference (WC) ≥ 80 cm, and 70.07% of men had WC ≥ 94 cm, suggesting an increased or highly increased risk of metabolic complications. More than half (51.22%) of patients were not physically active for more than 30 min every day, yet 85.56% of patients reported to eat vegetables and fruit every day; 51.92% took medications for high blood pressure on a regular basis; 12% of patients had high blood glucose at least once; and 43.05% of patients had family history of diabetes. Overall, 1685 patients had a high or very high risk of diabetes, according to FINDRISK (24.05%; 95% CI, 23.05–25.05%) [48].

In another study, a multi-level and multidisciplinary approach to improve diabetes control was implemented in a group of pharmacies for 10 months, between September 2016 and July 2017. Pharmacists in 26 pharmacies used FINDRISK questionnaire in 196 patients: 49.5% presented very high, high and moderate risk of diabetes. Of these, 23% were referred to the physician, nutrition or podiatric. Pharmacists in 31 pharmacies followed 106 diabetic patients, 52.8% of which were female, with an average age of 73.3 (SD = 8.4) years and having received an average of 9.2 medicines (SD = 3.4). Patients were referred to: nutrition and diabetic foot services (29.3%); follow-up in the pharmacy (25.0%); and to the physician (24%). Nurses conducted 3649 diabetic foot consultations, and nutritionists provided advice and follow-up to more than 180 people who specifically sought the nutrition service for diabetes control [49].

In 2016, with a new Government, the policy for SMBG products was revised, and a cap of 200 strips per type 2 diabetic patient not on insulin per year was defined for state reimbursement purposes. However, the policy of unregulated margins for product dispensing in pharmacies was kept [50].

Later that year, an important legislation was enacted following the First Agreement [51]. It established the terms for pharmacy-based public health interventions and incentives for dispensing reimbursed medicines of reference price groups. This legislation predicted that the Ministry of Health could contract public health interventions with pharmacies, if proven to bring an added value. It no longer specified diabetes self-monitoring, but integrated programs with primary care, collaboration in health technology assessment, and adherence monitoring, amongst others [51].

In 2016, Felix et al. estimated the social and economic benefits of Portuguese pharmacy public health interventions based on volume data of services provided in the last 25 years [52]. The annual economic value of promoting adherence, although not limited to diabetes, was estimated at 237.6 million euros, and that of direct interventions in diabetes was estimated at 32.9 million euros (15.8% of all pharmacy-based disease interventions). For diabetes, 286,186 patients were estimated to have a decrease in HbA1c of 0.7 percentage points. The estimated social value achieved a 4.7% gain in QoL and 10,707 additional quality-adjusted life-years (QALYs). The utilization of pharmacy services was estimated to prevent 274,577 physician visits, 2615 emergency-room visits, and 2615 hospitalizations [52]. These results represent the summary evidence derived from nationwide diabetes public health interventions in Portuguese pharmacies throughout the years.

In 2017, following legislation enacted in 2016, the Second Framework Agreement for Community Pharmacies was signed with the Ministries of Finance and Health [53].

Finally, in 2018, the scope of pharmacy health interventions was revised. The list of new interventions in pharmacies included the provision of level I care in the prevention and treatment of diabetic foot, according to guidance of the Directorate General for Health of the Ministry of Health [54].

## Discussion

Evidence exists on Portuguese pharmacy-based diabetes interventions throughout the years, as well as health policies adopted for that purpose. Some consistency has been observed in favorable outcomes stemming from those measures, but there is room for improvement considering policies, intervention design, and research methods.

The present review aimed to include all relevant published and unpublished data on the subject and tried to be the most inclusive as possible by not restricting comparators or outcomes.

Although comprehensive, the review has only included studies containing the selected search terms. Consequently, the authors admit that some data from relevant studies may have been missed. For instance, the review excluded manual search of specialized journals, hence data from those sources was not included.

Overall, findings from this review are consistent with those described in other jurisdictions.

The Portuguese health system is Beveridge type. It has public funding through taxes for the NHS with the following features: universal access; general coverage; tends to be free at the point of delivery (with some co-payment to moderate access); health services are provided by state services or commissioned to public or private entities. There are also private sub-systems.

Financing of medicines (and SMBG products) occurs through reimbursement by the NHS with some co-payment, depending on the ATC class of reimbursed medicine. Brand medicines with generics are grouped into reference cluster groups and reimbursement price is set at cluster level.

Portuguese pharmacies are private, financed by owners themselves, as in most European countries.

Despite the positive outcomes associated with Portuguese pharmacy-based diabetes interventions, the first capitated payment experience for pharmacies to provide diabetes management services has ended. This may hinder the large and mostly unexplored potential of pharmacies to function as a network of qualified health care providers towards diabetes care interventions in collaboration with primary care.

Contrarily to what happened in most countries, until 2016 there were no restrictions in Portugal for SMBG strip prescription for type 2 diabetic patients not on insulin. This may have contributed to the increase in NHS spending on SMBG strips over the years.

The remuneration for pharmacies to purchase, stock, and dispense reimbursed SMBG products is not regulated in Portugal, differing from the existing legal framework of regulated margins for reimbursed medicines. A proposal for a new pricing and margin methodology was considered, but never developed. Additionally, the legislation on patient’s right to choose a brand within INN prescribing regarding SMBG strips was also never established. Altogether, this tends to create an uneven market balance, which may further hinder pharmacies’ potential for improving self-monitoring adherence and cost control in collaborative care interventions with primary care.

Recently, in November 2017, the Gulbenkian Foundation launched an initiative to screen patients at risk of diabetes in 64 municipalities using FINDRISK. This initiative is still ongoing and, as of 1 May 2018, 383 pharmacies had screened 8112 individuals. From these, 4577 individuals at moderate, high or very high risk were referred to the physician, resulting in 190 patients with a confirmed diagnosed of diabetes (Source: Sifarma, ANF and SPMS, Ministry of Health / Analysis by CEFAR).

Finally, an adherence program to diabetic patients, including pharmacy on-site visits and scheduled refill text reminders, is one of the most recent projects occurring in Portuguese pharmacies.

Pharmacy-based diabetes interventions in other countries, currently remunerated by health payers and provided in large scale, have been investigated to retrieve common features to learn from.

Adherence reinforcement for patients new to type 2 diabetes medication was implemented in the UK through the New Medicines Service (NMS) [55]. Decision to remunerate this service was preceded by a trial showing a 10% adherence increase [56], and it was subsequently established as dominant (less costs, more benefits vs. usual care) [57]. Similar trials have been announced in Ireland and Switzerland.

In Australia, the 4th Community Pharmacy Agreement (CPA) 2005–2010 included funding for a diabetes management pilot [58], further reinforced by the 5th CPA [59]. In Alberta, Canada, the Government contracted with pharmacies to provide a Comprehensive Annual Care Plan for management of patients with two or more diseases, including diabetes [60].

Medicines Use Review is provided in the UK to patients at risk or diagnosed with cardiovascular disease and on ≥4 medications. Service is remunerated by the NHS per service [61]. A similar service is provided in Switzerland for patients on ≥4 medications for at least 3 months, reimbursed by health payer [62]. Diabetes MedsCheck is a similar service in Australia, funded by the 6th CPA [63]. Canada’s MedsCheck is funded in Ontario [60].

Screening individuals at risk and their referral is also part of the UK NHS Health Checks for people aged 40–74 years with no diagnosis of diabetes and no health check performed in the last 5 years. It is an Enhanced Service, locally commissioned in 38 geographical areas in the UK [64]. In Australia, the 6th CPA is currently funding a Pharmacy Trial Program (PTP) including diabetes screening [63].

Analysing the scope of these services and countries allows to retrieve important lessons.

All these countries have significantly changed their pharmacy remuneration system into an integrated system combining different components: dispensing, efficiency and/or quality incentives, and a small fraction for interventions related or not with dispensing.

All these countries have well-known, strong pharmacy organizations, which negotiated with payers and commissioned independent research to ascertain the value of interventions.

Both the scope of interventions and outcome measures are well defined and target populations have been narrowed to high-risk profiles to which pharmacies can add value. This seems to be the trend to restrain costs, along with payment systems per patient or cap payments. These are important features to consider in future studies aiming to improve health policies and practice.

Although these are complex health interventions, narrowing populations and defining scope of interventions and payment caps may facilitate their incorporation into routine dispensing and should be considered for large-scale implementation.

## Conclusions

### Implications for health policy and practice

There is some positive evidence stemming from pharmacy-based diabetes interventions in Portugal considering screening individuals at risk for the condition, monitoring diabetic patients, and referring patient to a physician when needed. There is also room for assisting patients in self-monitoring.

At the moment, there is no formal collaborative intervention between pharmacies and primary care regarding diabetes management, with shared incentives when improvements in patient outcomes are observed. There is, nonetheless, a window of opportunity in the framework of the legislation enacted in 2016 and the Second Framework Agreement for Community Pharmacy signed in 2017, with room for improvement in the development of integrated programs with primary care, in collaborating in health technology assessment, and in adherence monitoring.

Further directions can be explored considering integrated collaborations with primary care and adherence monitoring, with the potential do improve patient outcomes. This include screening of individuals at risk of diabetes and referral to the physician; counselling of new-to-therapy patients to improve adherence; adherence programs to patients on first-line metformin, with face-to-face visits and scheduled refill reminders; and medication therapy management to patients on multiple medications. Adherence programs for patients on first-line metformin, medication management for diabetic patients on multiple medications and assistance in self-monitoring may represent interesting priorities for future policies, with an impact on health outcomes and cost reduction.

Portuguese pharmacies could also perform intensive monitoring of new oral antidiabetic agents and retrieve associated real-world data, potentially relevant for the National Health Technology Assessment System for Portugal (SiNATS), created in 2015. Pharmacists collect relevant data on diabetic patients every day in their practice, which could be a major contribution for the development of patient registries and diabetic cohorts that could enable the analysis of safety, effectiveness, and quality of life outcomes. Such contribution should be valued in the regulatory setting, both for marketing conditional authorizations and health technology assessment. Post-safety and post-effectiveness authorization studies could benefit from this real-world evidence, with the potential to leverage the size and diversity of populations not included in clinical trials.

Pharmacists’ and physicians’ scientific and regulatory bodies should play a key role in providing evidence-based guidance for health professionals, defining standards for qualification and practice, while supporting a regulatory approach that boosts evidence-based practices aimed at contributing to the sustainability of the Health System.

### Implications for research

Future research in this area should prioritize the (cost)-effectiveness of experimental pharmacy-based diabetes programs in collaboration with primary care. Not all effective interventions are cost-effective. Hence, it is important to select interventions with the potential to bring this benefit to the society.

There is room for improvement in study design, namely through cluster RCTs or Pragmatic Controlled Trials (PCTs), which could explore other relevant outcomes, as PROs (for instance, by using validated, but short and feasible, scales for routine pharmacy practice).

Most interventions included in this review were not primarily designed as controlled trials. From a research perspective, RCTs are relevant as gold standard to establish efficacy. However, random patient selection is very difficult to achieve in real life. Cluster RCTs are recommended in public health interventions and could be used for a pilot trial, provided the choice of locations is guided by objective criteria and providers in the control arm are given the same training and tools to provide the intervention to patients at the end of the trial.

Finally, future research should also assess process indicators and other qualitative issues, as recommended for the evaluation of public health interventions.

## Additional file

Additional file 1: Search Strategy for Embase and MEDLINE (via Ovid). (DOC 23 kb)
