# Supplementary material for: Diabetes policies and pharmacy-based diabetes interventions in Portugal: a comprehensive review
Source: J Pharm Policy Pract. 2019 Mar 21;12:5. doi: 10.1186/s40545-019-0166-1 (PMC6427867; doi:10.1186/s40545-019-0166-1)
Supplement: Supplementary file 1 — Search Strategy for Embase and MEDLINE (via Ovid). (DOC 23 kb) [file 40545_2019_166_MOESM1_ESM.doc]

**Additional file – Search Strategy for Embase and MEDLINE (via Ovid)**

Embase <1996 to 2017 Week 46>, Ovid MEDLINE(R) Epub Ahead of Print, In-Process & Other Non-Indexed Citations, Ovid MEDLINE(R) Daily, Ovid MEDLINE and Versions(R).

--------------------------------------------------------------------------------

1 (pharmacy or pharmacist or pharmaceutical).ab,hw,kf,kw,ot,ti,tw.

2 (diabetes and portugal).ab,hw,kf,kw,ot,ti,tw.

3 1 and 2

4 Hospital.ab,hw,kf,kw,ot,ti,tw.

5 3 not 4

6 remove duplicates from 5

***************************
